# Prevalence of excessive daytime sleepiness among patients with atopic dermatitis

**DOI:** 10.1002/ski2.227

**Published:** 2023-03-21

**Authors:** Bruno Halioua, Jonathan Taieb, Julien Seneschal, Florence Corgibet, Laurent Misery, Adrien Marquié, Stéphanie Merhand, Delphine Staumont‐Salle, Khaled Ezzedine, Charles Taieb, Marie‐Aleth Richard

**Affiliations:** ^1^ Dermatologist Private Practice Paris France; ^2^ French Society of Human Skin Sciences (SFSHP) Maison de la dermatologie Paris France; ^3^ APHP Hôtel Dieu Centre du Sommeil et de la Vigilance Paris France; ^4^ Department of Dermatology and Pediatric Dermatology National Reference Center for Rare Skin Disorders Hospital Saint‐André Bordeaux France; ^5^ Dermatologist Private Practice Dijon France; ^6^ Department of Dermatology Brest University Hospital Brest France; ^7^ Data Analysis Paris France; ^8^ Association Francaise de l’Eczema Redon France; ^9^ Service de Dermatologie CHU Lille INFINITE U1286 Inserm University Lille Lille France; ^10^ EA 7379 EpidermE Université Paris‐Est Créteil (UPEC) Créteil France; ^11^ Patients Priority Department European Market Maintenance Assessment Fontenay‐sous‐Bois France; ^12^ Department of Dermatology Aix‐Marseille University La Timone University Hospital Marseille France; ^13^ Dermatology Department CEReSS‐EA 3279 Health Services and Quality of Life Research Centre Aix Marseille University La Timone University Hospital APHM Marseille France

## Abstract

Sleep disorders have received considerable attention from the dermatologic community, especially in patients with atopic dermatitis. We confirmed that excessive daytime sleepiness is a common problem among patients with atopic dermatitis, with it affecting 46.1% of the evaluated subjects. We demonstrated that excessive daytime sleepiness was also significantly associated with disease severity in patients with atopic dermatitis and had a detrimental impact on quality of life, well‐being and burden. These findings suggest the importance of careful assessment and the management of sleep disorders in atopic dermatitis patients. Intervention programs for sleep disorders in this population might help to improve their quality of life and their well‐being.

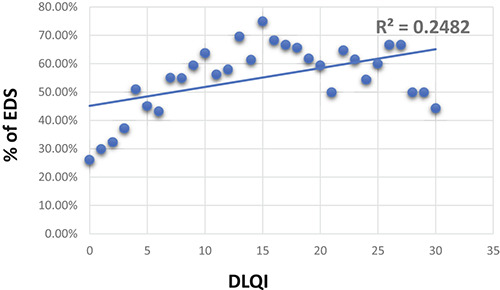

1

Dear Editor, Sleep disorders have received considerable attention from the dermatologic community, especially in patients with atopic dermatitis (AD).^1^ The prevalence of sleep complaints is very high in adults with AD, reportedly ranging from 33% to 87.1%.^2^ Excessive daytime sleepiness is a major consequence of sleep disorders with serious consequences both for individuals and society since it can increase the ratio of motor vehicle accidents, work‐related incidents, and deaths.^3^, ^4^ For example, a recent study reported that subjects with atopic dermatitis were 2.09 times more likely to have excessive daytime sleepiness than subjects without atopic dermatitis.^5^ Little is known about the excessive daytime sleepiness that occurs in adult patients with atopic dermatitis. The objective of our study was to assess the prevalence of excessive daytime sleepiness and to evaluate the associated clinical factors in patients with atopic dermatitis.

This was an observational, cross‐sectional study. The study was approved by local ethics committees at CHU de Grenoble, France (reference number 20.04.18.61210) and was conducted according to the principles of the Declaration of Helsinki.

Survey participants were recruited between January and April 2021, from a sample of French adults recruited by a polling institute (HC Conseil Paris, France) from the general adult population above 18 years of age using stratified, proportional sampling with a replacement design. Consecutive sampling is considered the best of the nonprobability sampling methods at controlling sampling bias because it includes all available subjects. Respondents who reported being diagnosed with AD by a physician were invited to participate in the study, and they were included if they're able to understand French; provided consent to participate in the study; and aged more than 18 years.

The questionnaire was constructed and endorsed by patient members of the French Eczema Association.

All participants answered a web‐based questionnaire on sociodemographic characteristics and subjective evaluations of sleep quality attributable or not to atopic dermatitis.

Objective clinical severity was assessed by the patient using the Patient‐Oriented Eczema Measure (POEM).^6^ POEM is a self‐assessment tool used to monitor disease activity in children and adults with AD. The questionnaire asks about the frequency of occurrence of 7 symptoms during the preceding week (itching, sleep, bleeding, weeping, skin cracking, skin flaking off, and skin dryness). It is rated out of 28. We used the previously proposed banding for POEM scores to create three groups: mild (0–7), moderate (8–16), and severe (17–28).^7^

In addition, patients were asked to complete 2 generic quality of life questionnaires (Short Form‐12 (SF‐12), Dermatology Quality of Life Index (DLQI) and an atopic dermatitis ‐specific burden questionnaire, the ABS‐A. specifically developed to evaluate the burden of atopic dermatitis in daily life.^8^

The DLQI is a self‐administered questionnaire that measures QOL over the past week in patients with skin disease. Scores range from 0 to 30, where a high score is equal to diminished quality of life.^9^ The SF‐12 is a short version of the Short Form‐36 (SF‐36), a generic measure enabling assessment of health status in the general population.^10^ Two scores, the Physical Component Summary (PCS‐12) and the Mental Component Summary (MCS‐12), can be calculated from the 12 questions of the SF‐12. There is no overall score. A higher score indicates a better quality of life. All participants were asked to complete three questionnaires. The Epworth Sleepiness Scale (ESS) contains 8 questions, and the scores range from 0 to 24.^11^ Excessive daytime sleepiness was defined by an Epworth score ≥10.^12^

In general, a score greater than 10 was defined as excessive daytime sleepiness. The WBQ12 is a reliable and valid measure of well‐being with three 4‐item subscales: Negative Well‐Being (NWB), Energy (ENE), and Positive Well‐Being (PWB). Items are rated on a 4‐point Likert‐scale ranging from 3 (“all the time”) to 0 (“not at all”).^13^

French validation of these questionnaires was used.

The intensity of itch, burn sensation and pain was evaluated with a visual analog scale (VAS) as the mean symptom experienced within the last 3 days (VAS mean) and at the time of examination (VAS exam). Scores ranged from 0 (no itch/burn sensation/pain) to 10 points (worst itch/burn sensation/pain imaginable). A chi‐square test and Fisher's exact test were used to compare differences in categorical data, and an independent *t* test was used for continuous variables. Correlations between tested parameters were verified with the Spearman rank correlation test. A value lower than 0.05 was considered statistically significant.

Among the 2530 participants in the study, 1561 were female (61.7%), and 969 were male (38.3%) (mean ± standard deviation (SD) age 42.4 ± 13.9 years). Of these patients, 1236 (48.9%; 44.9 ± 14.3 years) had mild atopic dermatitis, and 1294 (51.1%; 39.9 ± 13.1 years) had moderate and severe atopic dermatitis (*p* < 0.001).

The prevalence of excessive daytime sleepiness was 46.1% (1167/2530), without significant difference in women (45% 702/1561) and in men (48% 465/969). However, prevalence of Excessive daytime sleepiness was significantly more frequent in patient aged ≤ 45 ans (796/1573 [50.6%] vs. 371/957 [38.8%] (*p* < 0.001).

Among people with excessive daytime sleepiness, 58.4% felt tired, exhausted or worn out all the time, 50.1% often, 42.1% sometimes and 25.1% never.

The imputability of atopic dermatitis was more frequently reported in patients with excessive daytime sleepiness (47.2% vs. 32.9% (*p* < 0.05)). Patients with excessive daytime sleepiness reported shorter nighttime sleep duration (6.51 ± 1.53 vs. 6.92 ± 1.46 *p* < 0.05) and getting less than 6 h of sleep a night at least 3 times a week (64.4% vs. 46.2% *p* < 0.05). A total of 46.2% of patients with excessive daytime sleepiness still exclusively reported sleep‐onset insomnia (SOI), 51.2% exclusively reported sleep maintenance insomnia (SMI), and 47.4% reported SOI associated with SMI.

Patients with excessive daytime sleepiness more frequently presented moderate and severe disease (61.4% vs. 42.4%, *p* < 0.05) and showed a significantly higher impact on quality of life and burden and lower well‐being (*p* < 0.05; Table 1). A significant correlation was noted between DLQI and prevalence of excessive daytime sleepiness R: 0.482, *p* < 0.01; Figure 1).

**TABLE 1:** Assessment burden, alterations in quality of life, well‐being and cutaneous symptomatology in patients with atopic dermatitis (AD) with regard to EDS.

	Presence of excessive daytime sleepiness	Absence of excessive daytime sleepiness	
	Mean ± SD	Mean ± SD	
Age	40.35 ± 13.41	44.09 ± 14.13	<0.0001
PCS12	46.64 ± 7.41	49.36 ± 7.27	<0.0001
MCS12	44.15 ± 6.97	46.66 ± 6.97	<0.0001
BURDEN	40.34 ± 17.40	30.99 ± 16.91	<0.0001
DLQI	8.82 ± 6.75	5.56 ± 6.19	<0.0001
WB‐Q12	17.73 *±* 5.89	20.63 ± 6.88	<0.0001
Itching	5.3 ± 2.27	4.44 ± 2.53	<0.0001
Skin pain	4.36 ± 2.59	3.26 ± 2.70	<0.0001
Burning sensation	4.33 ± 2.71	3.19 ± 2.78	<0.0001

Abbreviations: ABS‐A, Atopy Burden Score – Adult; DLQI, dermatology quality of life index; MCS, mental component summary; PCS, physical component summary; SF, short form.

**FIGURE 1:**
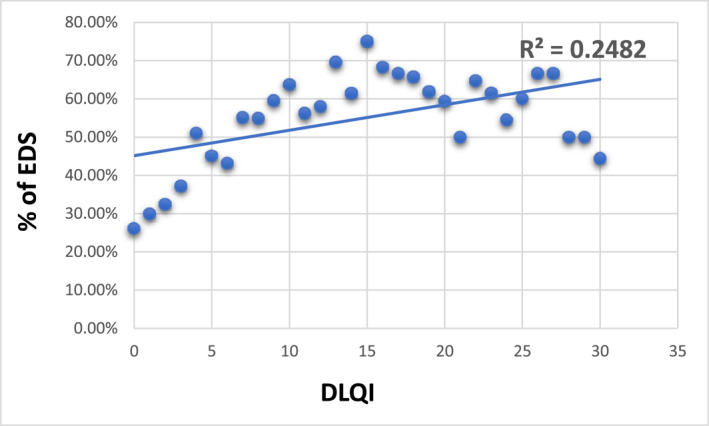
Correlation between DLQI and prevalence of excessive daytime sleepiness.

Complaining about excessive daytime sleepiness was significantly associated with a high intensity of pruritus, cutaneous pain and burn sensation (*p* < 0.05; Table 1).

To the best of our knowledge, this study has performed the most comprehensive evaluation of excessive daytime sleepiness in a population of patients with atopic dermatitis. A U.S. study conducted by the National Health Interview Survey previously found that eczema was associated with a higher likelihood of regular daytime sleepiness.^14^ We confirmed that excessive daytime sleepiness is a common problem among patients with atopic dermatitis, with affecting 46.1% of the evaluated subjects. We observed that fatigue was more common among AD patients with excessive daytime sleepiness than among those without excessive daytime sleepiness. Nighttime sleep problems are significant predictors of excessive daytime sleepiness. We demonstrated that excessive daytime sleepiness was also significantly associated with disease severity in patients with atopic dermatitis and had a detrimental impact on quality of life, well‐being and burden. We know, optimal management of excessive daytime sleepiness in AD requires effective therapy for sleep disorders and symptoms including pruritus, cutaneous pain and burn sensation.^15^ These findings suggest the importance of careful assessment and the management of sleep disorders in atopic dermatitis patients. Intervention programs for sleep disorders in this population might help to improve their quality of life and their well‐being.

Our results provide a basis for future study, and, perhaps most importantly, suggest new avenues for interventions aimed at improving excessive daytime sleepiness in individuals with DA.

## CONFLICT OF INTEREST STATEMENT

None to declare.

## AUTHOR CONTRIBUTIONS

**Bruno Halioua**: Methodology (Equal); Writing – original draft (Lead); Writing – review & editing (Lead). **Jonathan Taieb**: Validation (Equal); Writing – original draft (Equal). **Julien Seneschal**: Conceptualization (Equal); Methodology (Equal); Writing – original draft (Equal). **Florence Corgibet**: Methodology (Equal); Writing – original draft (Equal). **Laurent Misery**: Conceptualization (Equal); Methodology (Equal); Writing – original draft (Equal). **Adrien Marquie**: Formal analysis (Equal). **Stéphanie Merhand**: Conceptualization (Equal); Funding acquisition (Equal); Methodology (Equal); Writing – original draft (Equal). **Delphine Staumont‐Salle**: Conceptualization (Equal); Methodology (Equal); Writing – original draft (Equal). **Khaled Ezzedine**: Conceptualization (Equal); Methodology (Equal); Writing – original draft (Equal). **Charles Taieb**: Conceptualization (Equal); Formal analysis (Equal); Investigation (Equal); Methodology (Equal); Writing – original draft (Equal); Writing – review & editing (Equal). **Marie‐Aleth Richard**: Conceptualization (Equal); Methodology (Equal); Writing – original draft (Equal).

2

## ETHICS STATEMENT

The study was approved by local ethics committees at CHU de Grenoble, France (reference number 20.04.18.61210) and was conducted according to the principles of the Declaration of Helsinki.

## Data Availability

The data that support the findings of this study are available from the corresponding author upon reasonable request.
